# Prioritizing breast cancer surgeries: insights from the KRONOS SCORE

**DOI:** 10.3389/fonc.2024.1465154

**Published:** 2025-01-21

**Authors:** Lucía Navarro, Luis Perez-Bartivas, David Ponferrada, Javier Cejas, Juan Adrián Camús, Natalia Rangel, Maria Porras, Cristina Morales Estevez, Amalia Palacios Eito, Pilar Rioja, Marina Alvarez, Enrique Aranda, Juan De La Haba-Rodríguez

**Affiliations:** ^1^ Medical Oncology Department, University Hospital Reina Sofia, Cordoba, Spain; ^2^ Maimonides Biomedical Research Institute of Cordoba (IMIBIC), Cordoba, Spain; ^3^ Medical School, University of Cordoba, Cordoba, Spain; ^4^ Breast Cancer Surgery Department, University Hospital Reina Sofia, Cordoba, Spain; ^5^ Radiotherapy Oncology Department, University Hospital Reina Sofia, Cordoba, Spain; ^6^ Breast Cancer Radiology Department, University Hospital Reina Sofia, Cordoba, Spain

**Keywords:** breast cancer, node staging, clinical outcomes, time to surgery, lymph node metastasis, neoplasm grading

## Abstract

**Introduction:**

In many healthcare systems, the time to surgery (TTS) is used as a quality measure in breast cancer (BC) care. Although guidelines suggest that a waiting period of up to four weeks is acceptable, this is often exceeded, potentially impacting treatment outcomes. The COVID-19 pandemic highlighted the need to reassess surgical urgency. This study aims to explore the relationship between TTS and clinical outcomes in BC patients, focusing on how TTS influences final tumor stage and lymph node involvement.

**Materials and methods:**

A retrospective cohort study was conducted, including 924 women diagnosed with cT1-T3 BC without axillary lymph node involvement between 2014 and 2023. Preoperative staging was done using mammography and ultrasound, while postoperative staging relied on definitive anatomopathological reports. Statistical analyses included chi-square tests, Wilcoxon tests, and binary logistic regression. A prognostic score, the KRONOS SCORE, was developed based on significant variables from the final model, and internal validation was performed.

**Results:**

Out of 924 patients, 781 had ductal carcinomas, 127 had lobular carcinomas, and 16 had other histologies. Breast-conserving surgery was performed on 664 patients, while 260 underwent mastectomy. TTS was less than 8 weeks for 513 patients and more than 8 weeks for 411 patients. No significant differences in tumor size changes were observed based on TTS. However, lymph node involvement increased from 23.8% in patients operated on within 8 weeks to 26.5% in those operated on after 8 weeks, with significant differences noted in poorly differentiated tumors (G3). The KRONOS SCORE, based on age, tumor grade, and size, was validated, showing a higher risk of lymph node involvement with higher scores.

**Discussion:**

Although TTS did not significantly affect most clinical and pathological parameters, a trend towards increased lymph node involvement with prolonged TTS was observed, particularly in patients with poorly differentiated tumors (G3). The KRONOS SCORE offers a tool for prioritizing surgical patients based on risk factors. However, further multicenter and prospective studies are needed to validate these findings and the KRONOS SCORE model’s effectiveness in different clinical settings.

## 1 Introduction

In numerous healthcare systems, TTS has been incorporated as a quality measure in the care process for BC (1, 2). Although various guidelines suggest that a waiting period of up to four weeks is acceptable, in practice, this period is frequently extended in many healthcare systems, which could adversely affect treatment outcomes (3).

The challenges imposed by health emergencies, such as the COVID-19 pandemic, have exacerbated the need to reassess which patients require more urgent interventions and which can tolerate longer waiting periods without a deterioration in prognosis (4). Our study aims to fill existing knowledge gaps by providing a more detailed and updated perspective on the relationship between surgical time and clinical outcomes in patients with breast cancer.

Despite efforts to standardize waiting times for breast cancer surgery, the results highlight contradictory findings that do not always consider the clinicopathological characteristics of the tumor, suggesting the need for a more personalized approach (**Table 1**).

**Table 1:** Studies analyzing the prognostic value of time to surgery in breast cancer.

Study	No. of Patients	Time to Surgery	Key Results
Eaglehouse et al., 2019 (5)	9,669	0, 1-21, 22-35, >36 days	Overall mortality HR increases with times greater than 36 days.
Smith et al., 2013 (6)	8,860	>6 weeks, <2 weeks	5-year survival is lower in >6 weeks (80% vs. 90%).
Bleicher et al., 2016 (7)	94,544	<30, 31-60, 61-90, 91-120, 121-180 days	BC-specific mortality increases with each 60-day interval.
Beaubrun-Renard et al., 2023 (8)	713	<1 month, 1-2 months, 2-4 months, >4 months	Better OS in 1-2 months compared to >4 months (HR 3.1).
Eriksson et al., 2018 (9)	7,017	>3 weeks, >6 weeks	Increased HR in surgery after 6 weeks.
Zhu et al., 2023 (10)	5,130	<1 week, 1-2 weeks, >2 weeks	Shorter BC-free interval and OS in >2 weeks.
Wiener et al., 2023 (4)	373,334	0-4 weeks, >8 weeks	Worse OS in >9 weeks compared to 0-4 weeks (HR 1.15).

Explanation of terms: HR, Hazard Ratio; OS, Overall Survival; BC, Breast Cancer.

This table summarizes the results of various studies evaluating the impact of the time interval to surgery on the prognosis of breast cancer patients.

This study specifically focuses on investigating how the time to surgery can alter the final tumor stage in patients with BC, examining how clinical and histobiological factors may influence this process.

## 2 Materials and methods

### 2.1 Study design

A longitudinal, analytical, and observational retrospective cohort study was conducted, including women diagnosed with invasive breast carcinoma where the first therapeutic strategy performed was surgery.

### 2.2 Study population

The study included 924 patients diagnosed between 2014 and 2023 with cT1-T3 breast cancer without axillary lymph node involvement, confirmed by axillary ultrasound. Patients whose initial therapeutic strategy was neoadjuvant treatment or who were diagnosed with bilateral breast cancer were excluded from the study.

### 2.3 Diagnostic methods

For preoperative staging, mammography and axillary ultrasound data were collected. Cases with lymph node involvement were excluded following the ultrasound criteria established in the literature (11). Postoperative staging was based on the data from the definitive anatomopathological report.

### 2.4 Statistical method

#### 2.4.1 Univariable and multivariable analysis

The chi-square test was used to analyze associations between nominal qualitative variables, and the Wilcoxon test was used for ordinal qualitative variables. Binary logistic regression was employed, with postoperative lymph node involvement as the outcome variable. Variables with a p-value greater than 0.15 were progressively eliminated from the model based on the Wald statistic, while interactions and confounding factors were examined in variables with p-values between 0.05 and 0.15. Variables with p-values less than 0.05 were included in the final model.

#### 2.4.2 Model validation

The Hosmer-Lemeshow test was performed to evaluate the validity and fit of the statistical model.

#### 2.4.3 Score development

Based on the odds ratios of the significant variables in the final model, a prognostic score was designed. This score was calculated for each patient, establishing three risk groups according to the obtained score.

#### 2.4.4 Prognostic group analysis

The chi-square test was again applied to determine statistically significant differences in the risk of lymph node progression between the groups defined by the score.

#### 2.4.5 Statistical significance

Statistical significance was defined with a p-value less than 0.05 and a beta error less than 0.2.

#### 2.4.6 Statistical software

IBM SPSS Statistics version 28^®^ was used for all statistical analyses.

## 3 Results

A total of 924 patients without axillary lymph node involvement on ultrasound were included, of which 781 (84.7%) had ductal carcinomas, 127 (13.8%) had lobular carcinomas, and 16 (1.5%) had other histologies. Regarding surgical intervention, 664 patients (71.9%) underwent breast-conserving surgery, while 260 (28.1%) were treated with mastectomy.

The distribution of time to surgery showed that 513 patients (55.5%) were operated on before 8 weeks and 411 (44.5%) after 8 weeks. The distribution of clinicopathological characteristics and their statistical significance based on the time to surgery is shown in **Table 2**.

**Table 2:** Distribution of clinicopathological characteristics and their statistical significance based on time to surgery.

Characteristic	0-8 weeks (n=513)	>8 weeks (n=411)	p-value
Tumor Size (Mammographic)			
T1	298 (65.2%)	236 (62.4%)	0.132
T2	138 (30.2%)	112 (29.6%)	
T3	21 (4.6%)	30 (7.9%)	
Tumor Grade			
G1	158 (30.8%)	139 (33.8%)	0.618
G2	240 (46.8%)	183 (44.5%)	
G3	115 (22.4%)	89 (21.7%)	
Estrogen Receptor (ER)			
Positive	475 (92.6%)	388 (94.4%)	0.204
Negative	38 (7.4%)	23 (5.6%)	
Progesterone Receptor (PR)			
Positive	420 (81.9%)	338 (82.2%)	0.323
Negative	93 (18.1%)	73 (17.8%)	
HER2			
Positive	29 (5.7%)	27 (6.6%)	0.655
Negative	484 (94.3%)	384 (93.7%)	

This table presents the distribution of various clinicopathological characteristics of breast cancer patients based on whether they had surgery within 0-8 weeks or after 8 weeks. The p-values indicate the statistical significance of differences between the two groups for each characteristic.

### 3.1 Analysis of tumor size modification and lymph node involvement

**Figure 1:**
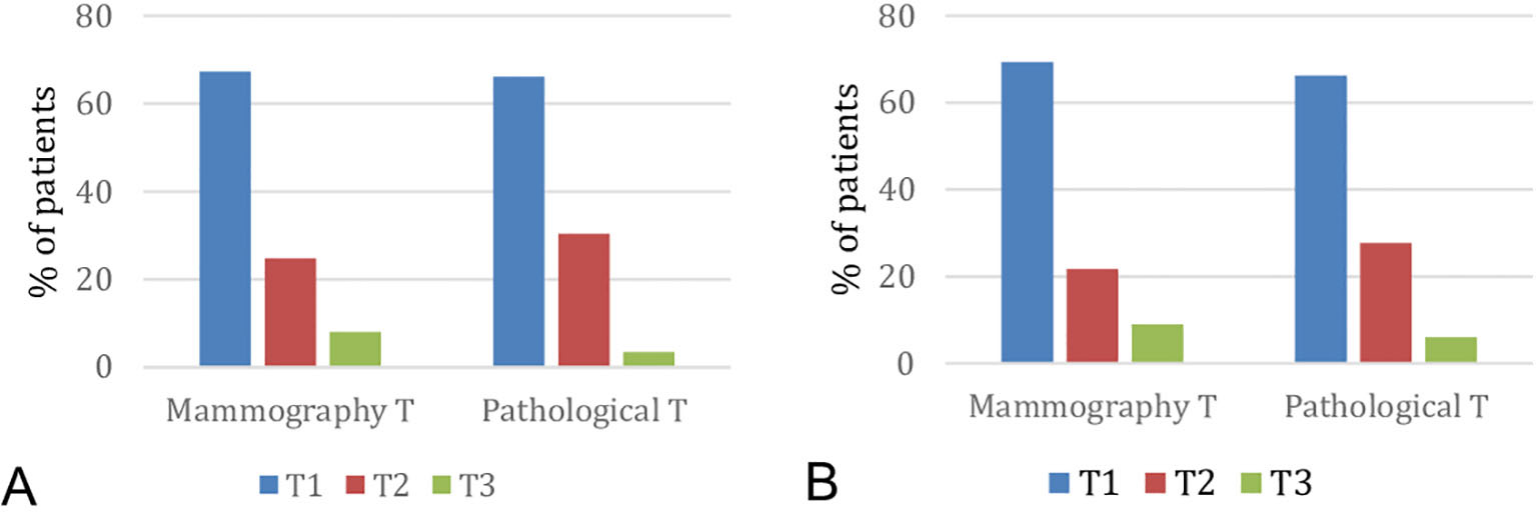
**(A)** Comparison of pathological size with mammographic size in patients who underwent surgery before 8 weeks (p=0.11). **(B)** Comparison of pathological size with mammographic size in patients undergoing surgery after 8 weeks (p=0.981).

The analysis of preoperative tumor size in relation to postoperative pathological size showed no statistically significant differences in patients operated on before 8 weeks (p=0.11) (**Figure 1A**) or in those operated on after 8 weeks (p=0.981) (**Figure 1B**).

**Figure 2:**
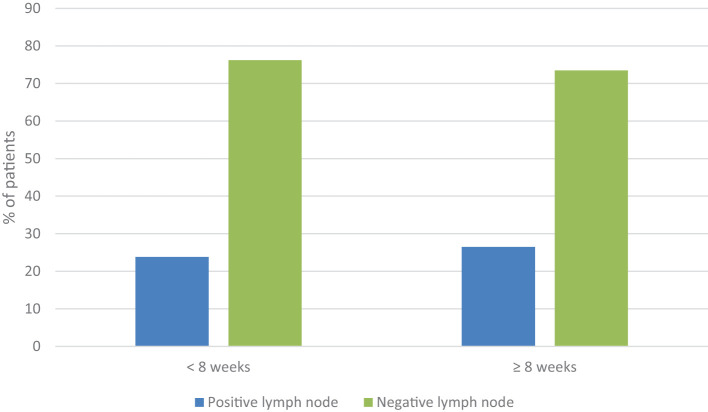
Change in staging from N0 to N+ post-surgery in patients who underwent surgery before 8 weeks compared to those who underwent surgery after 8 weeks (p=0.339).

Regarding lymph node involvement, 23.8% (122 patients) of those operated on before 8 weeks experienced a change in their postoperative staging to N+, compared to 26.5% (109 patients) of those operated on after 8 weeks, without statistically significant differences (p=0.339) (**Figure 2**).

### 3.2 Influence of biological factors

**Figure 3:**
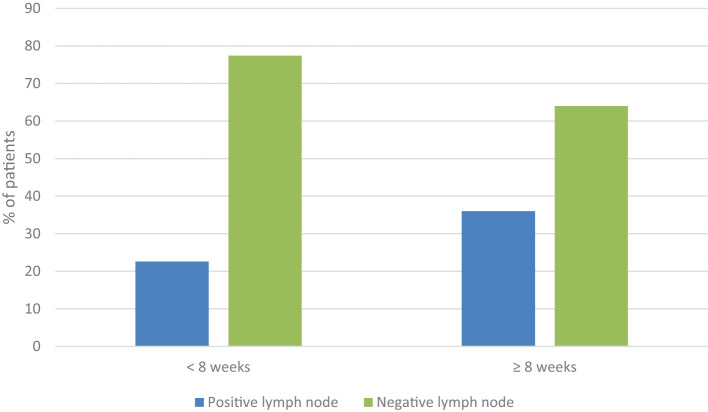
Change in staging from N0 to N+ post-surgery in patients who underwent surgery before 8 weeks compared to those who underwent surgery after 8 weeks as a function of the degree of differentiation (G3) (p= 0.036).

No statistically significant differences were found in the change of tumor size based on the biological factors studied. However, of the 115 patients with G3 tumors operated on before 8 weeks, 26 (22.6%) changed their postoperative staging to N+, while 89 patients (77.4%) maintained their preoperative N0 staging. Among the 89 patients operated on after 8 weeks, 32 (36%) changed their postoperative staging to N+ while 57 patients (64%) maintained their preoperative N0 staging. The increase in postoperative lymph node involvement in patients operated on after 8 weeks shows statistically significant differences (p=0.036) compared to patients operated on within the first 8 weeks (**Figure 3**).

These results suggest that while the time to surgery did not significantly influence most of the clinical and pathological parameters evaluated, it could have an effect on the progression of patients with certain advanced histological and biological characteristics.

### 3.3 KRONOS SCORE

To develop the KRONOS SCORE, patients with tumors lacking hormone receptor expression (HR-) were excluded due to their low representation in the sample, approximately 7%, which is lower than generally reported in the literature. This is because most HR- tumors are managed with neoadjuvant chemotherapy, which was an exclusion criterion for this study since the first therapeutic strategy had to be surgical.

In the univariate analysis, sex, age, histology, tumor grade, HER2 expression, the proliferation marker Ki67, time to surgery (less or more than 8 weeks), and tumor size detected on mammography were included as covariates. Variables that showed statistical and clinical significance (p<0.2) advanced to multivariate analysis. After adjusting for confounding factors and interactions, the variables that maintained statistical significance were age, tumor grade, and tumor size. The results of the univariate and multivariate analysis are presented in **Table 3**.

**Table 3:** Univariate and multivariate analysis of the KRONOS SCORE.

Variables	Univariate Analysis OR (95% CI)	p-value	Multivariate Analysis OR (95% CI)	p-value
SEX	0.485 (0.058-4.049)	0.504		
Age >60	0.730 (0.536-0.994)	0.046	0.695 (0.507-0.955)	0.025
Grade				
G2 vs G1	2.381 (1.626-3.486)	0.000	2.245 (1.526-3.304)	0.000
G3 vs G1	2.725 (1.722-4.314)	0.000	2.448 (1.531-3.915)	0.000
Tumor Size				
T2 vs T1	2.035 (1.432-2.893)	0.000	1.821 (1.268-2.614)	0.001
T3 vs T1	1.709 (1.057-3.088)	0.030	1.672 (0.968-2.887)	0.065

This table shows the odds ratios (OR) and 95% confidence intervals (CI) for both univariate and multivariate analyses of the variables included in the KRONOS SCORE. The p-values indicate the statistical significance of each variable in predicting the outcome.

Based on these data, the KRONOS SCORE was developed, with the variables having the greatest weight in the scale (**Table 4**):

- Diagnosis of breast cancer before 60 years (3 points)
- Moderately differentiated tumor grade (G2) (4 points)
- Poorly differentiated tumor grade (G3) (5 points)
- Preoperative tumor size cT>1 (4 points)

**Table 4:** KRONOS SCORE.

Variable	Score
Age under 60 years	3 points
Tumor grade	
Tumor grade G2	4 points
Tumor grade G3	5 points
Ultrasound/mammogram T > T1	4 points
MAXIMUM SCORE	12 POINTS

### 3.4 Internal validation of the KRONOS SCORE

An internal validation was conducted within our sample, stratifying into 3 groups according to the score obtained. The results reflect a gradual increase in the risk of lymph node involvement with higher scores. **Table 5** shows that patients with a score of 0-3 points (low risk) mostly maintained their N0 postoperative staging, while those with a high score (8-12 points) showed a significant increase in the probability of having postoperative lymph node involvement.

**Table 5:** Application of the KRONOS SCORE on the study sample as a method of internal validation (p<0.001).

	0-3 points n (%)	4-7 points n (%)	8-12 points n (%)
Negative lymph node	205 (87.6)	275 (73.5)	163 (63.9)
Positive lymph node	29 (12.4)	99 (26.5)	92 (36.1)

## 4 Discussion

The importance of delay in primary surgery for patients with early-stage BC has been widely studied, both in relation to its association with worse disease prognosis and its connection with care quality, being established as a marker of the latter. Recently, various studies have addressed how delay in the time to surgery affects patient survival; however, these studies often lack data describing the disease progression of these patients and its relationship with prognosis (7–10).

One of the key parameters for understanding to what extent the time to surgery influences disease progression is the interval of disease-free survival (DFS). This parameter is not included in most of the aforementioned studies, with the exception of a study published in Scientific Reports, which provides data on DFS (3). In this study, the time intervals to surgery evaluated were grouped into less than 1 week, 1-2 weeks, and more than 2 weeks. According to previous studies, this cutoff is not significant for evaluating changes in prognosis and, moreover, does not represent a realistic objective for current waiting lists (4, 12).

Regarding survival analysis, in the mentioned studies, it is measured with overall mortality data. There is no consensus on the time interval to surgery to be evaluated, which makes it difficult to establish a common study time interval between different projects.

However, in our study, we have considered pre- and postoperative lymph node involvement to study the progression of BC in patients operated on before and after 8 weeks. By using the change in postoperative staging as a surrogate marker, we can offer results that reflect disease progression instead of relying solely on survival data related to overall mortality, which can be affected by factors external to the disease.

Moreover, this approach allows us to evaluate the importance of time to surgery in BC. While, as expected given the evolutionary heterogeneity of the disease, no statistically significant results were found, the graphs reflect a marked trend toward increased lymph node involvement the longer the time to surgery, both globally and in certain biological subgroups, such as poorly differentiated tumors (G3).

Although the relevance of biological factors in BC progression is widely recognized, we have been able to directly verify their influence when establishing the time to surgery in certain patients and, therefore, prioritize some cases over others.

During the study, we analyzed important biological factors such as histological grade, hormone receptor expression, HER2, and the proliferation index Ki67. The influence of tumor grade has been extensively documented. However, concerning Ki67, we observed in **Table 3** that, although there are significant differences in the univariate analysis when comparing groups with higher proliferation indices, these are not observed when comparing the influence of the Ki67 proliferation index in the group of patients operated on after 8 weeks versus those operated on before 8 weeks. This is because, although this biological factor does not directly influence the time to surgery, it does have a clear relationship with tumor progression and prognosis, as reflected in our study.

With the results obtained in the multivariate analysis, we observed that age at diagnosis under 60 years, moderately differentiated histological grade (G2), poorly differentiated grade (G3), and preoperative tumor size cT>1 cm have a clinically and statistically significant influence on BC. Based on these data, a scoring model, the KRONOS SCORE, was developed, which is stratified into 3 groups from lower to higher risk of progression according to the score obtained.

This scoring system could be useful for organizing surgical waiting lists for HR+ patients, as there are currently no established criteria that consider the different characteristics of surgical candidates and their tumors in a weighted manner. This is reflected in **Table 2**, where no statistically significant differences were found for any of the biological factors included in the analysis of patients in this study.

With the KRONOS SCORE, we have the opportunity to reorganize these lists by developing software that prioritizes patients who may have a higher risk of disease progression during this wait.

This study also presents some limitations, as it is retrospective and single-center. The results, therefore, have not been evaluated in other centers to verify their reproducibility and the effectiveness of the predictive model. As previously explained, for the multivariate analysis, patients with HR- tumors were excluded from the study due to the lack of patients in this subgroup who had not received neoadjuvant treatment, so the score can only be applied to tumors with hormone receptor expression.

We can conclude that this study has shown that, although the time to surgery did not have a statistically significant impact on most of the clinical and pathological parameters evaluated, it does reveal a trend towards increased lymph node involvement with prolonged time to surgical intervention. This observation is particularly notable in patients with poorly differentiated histological tumors (G3), where extended time to surgery seems to negatively influence disease progression. The application of the KRONOS SCORE, developed in this study, can provide a potentially valuable tool for prioritizing patients on waiting lists based on biological and clinical risk factors that could more significantly affect disease prognosis. The implementation of this scoring model can help optimize the clinical management of breast cancer, especially in settings with limited resources or prolonged waiting times. However, further research is needed to validate these findings in multicenter and prospective studies to ensure the reproducibility and effectiveness of the KRONOS SCORE model in different populations and clinical contexts.

## Data Availability

The datasets presented in this study can be found in online repositories. The names of the repository/repositories and accession number(s) can be found in the article/supplementary material.
